# New developments on the Encyclopedia of DNA Elements (ENCODE) data portal

**DOI:** 10.1093/nar/gkz1062

**Published:** 2019-11-12

**Authors:** Yunhai Luo, Benjamin C Hitz, Idan Gabdank, Jason A Hilton, Meenakshi S Kagda, Bonita Lam, Zachary Myers, Paul Sud, Jennifer Jou, Khine Lin, Ulugbek K Baymuradov, Keenan Graham, Casey Litton, Stuart R Miyasato, J Seth Strattan, Otto Jolanki, Jin-Wook Lee, Forrest Y Tanaka, Philip Adenekan, Emma O’Neill, J Michael Cherry

**Affiliations:** Department of Genetics, Stanford University, Stanford, CA 94305-5477, USA

## Abstract

The Encyclopedia of DNA Elements (ENCODE) is an ongoing collaborative research project aimed at identifying all the functional elements in the human and mouse genomes. Data generated by the ENCODE consortium are freely accessible at the ENCODE portal (https://www.encodeproject.org/), which is developed and maintained by the ENCODE Data Coordinating Center (DCC). Since the initial portal release in 2013, the ENCODE DCC has updated the portal to make ENCODE data more findable, accessible, interoperable and reusable. Here, we report on recent updates, including new ENCODE data and assays, ENCODE uniform data processing pipelines, new visualization tools, a dataset cart feature, unrestricted public access to ENCODE data on the cloud (Amazon Web Services open data registry, https://registry.opendata.aws/encode-project/) and more comprehensive tutorials and documentation.

## INTRODUCTION

Over 99% of the human genome was sequenced as a part of the Human Genome Project that was completed in April 2003 (1). Learning the function of genomic sequences remains a challenge and even today the biological purpose of a large fraction of the human genome is largely unknown. The Encyclopedia of DNA Elements (ENCODE) project is a public research consortium funded by the National Human Genome Research Institute initiated after the completion of the Human Genome Project in 2003 (2). The goal of the ENCODE project is to identify all the functional elements in the human and mouse genomes. All experimental metadata, raw data and analysis results generated by the ENCODE project are freely accessible to the scientific community on the ENCODE portal (https://www.encodeproject.org/), developed and maintained by the ENCODE Data Coordinating Center (DCC) (3–6). In addition to the data generated by the ENCODE consortium, the ENCODE portal also hosts data from modENCODE (7), modERN (8), The NIH Roadmap Epigenomics Consortium (9) and Genomics of Gene Regulation (https://www.genome.gov/27561317/genomics-of-gene-regulation/) projects as well as some datasets provided by other members of the scientific community. The ENCODE portal provides unrestricted access to more than 15 000 experimental results from more than 40 different categories of high-throughput sequencing (HTS) technologies in over 75 different cell and tissue types. The ENCODE portal hosts more than 640 terabytes of data files and this is expected to grow to over 1 petabyte by 2021.

The ENCODE DCC is a critical component of the ENCODE project, working to maximize the accessibility and utility of the data generated by the consortium. Using the data model developed by the DCC, ENCODE production labs format and submit to the ENCODE portal structured metadata describing their experiments and the data generated by the experiments. In addition to serving as the centralized data deposition repository of the ENCODE consortium, the DCC is also responsible for primary data analysis of key ENCODE assays. The DCC processes the raw data using the ENCODE uniform processing pipelines and submits processed analysis files to the portal (3,4,6). The DCC is also responsible for the documentation of a variety of metadata and data standards developed by the ENCODE consortium, as well as for the implementation of automated checks to validate data against these standards (6). Collectively, these efforts ensure that ENCODE data are findable, accessible, interoperable and reusable (10) and follow the original metadata organization principles (6).

## DATA ON THE ENCODE PORTAL

**Figure 1.**
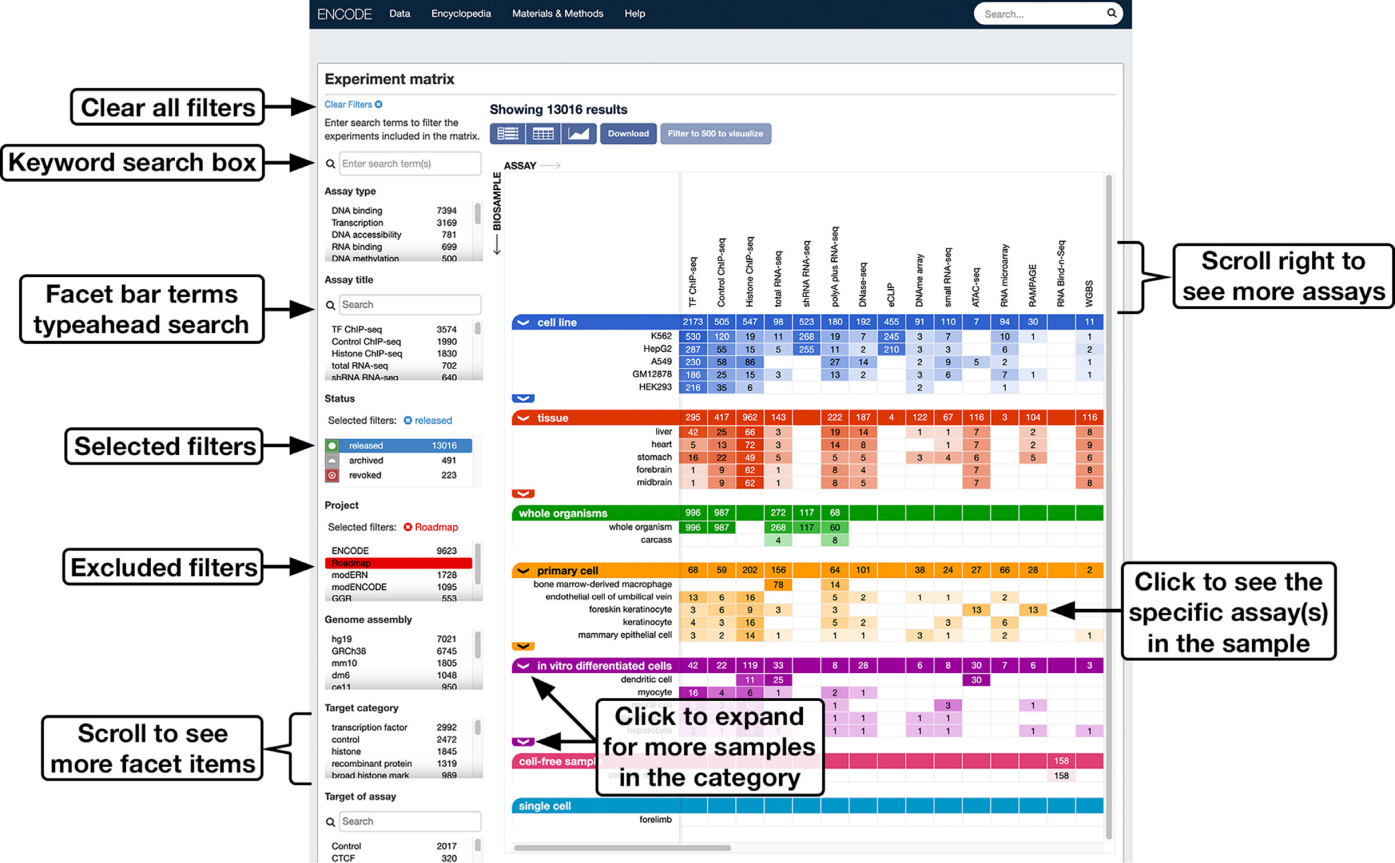
Experiment matrix. The ENCODE portal hosts data generated by more than 40 different biochemical assays, listed on the *x*-axis of the matrix. The *y*-axis lists various sample types represented on the portal. Each cell in the matrix indicates the number of experiments that are currently available on the portal with a particular sample type and the corresponding assay type. To the left of the matrix are faceted browsing interface bars that can be used to positively select (blue) and negate selection (red) of certain values of specific experimental metadata properties.

As an ongoing collaborative research project, both the size and the spectrum of the data available on the ENCODE portal continue to grow from year to year (3). More than 2000 experiments of various assay types were added to the portal in the past year, including experiments studying more than 30 new sample types (Supplementary Table S1). Data generated by new HTS assays and cutting-edge technologies have been made publicly available, including, but not limited to, single-nucleus ATAC-seq (11), icSHAPE (12) and long-read RNA-seq (13 Wyman, D. et al. bioRxiv, https://doi.org/10.1101/672931). To get a high-level overview of the available data on the portal, users can go to the experiment matrix page (https://www.encodeproject.org/matrix/?type=Experiment&status=released), which presents the experiments as a matrix with sample types listed as its *y*-axis and assay types as its *x*-axis. The matrix along with the faceted browsing interface on the left-hand side of the screen is designed to help users find experiments of interest (Figure 1).

## FACETED BROWSING INTERFACE

**Figure 2.**
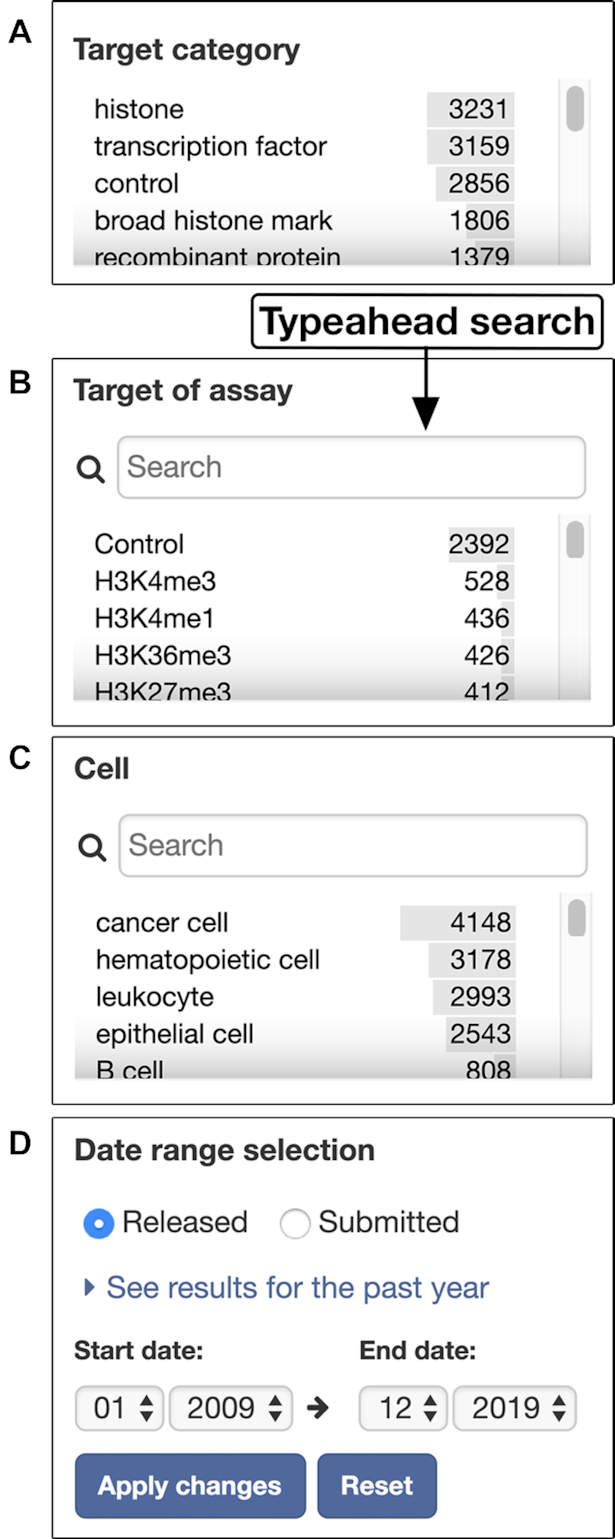
Faceted browsing interface. Four new facet bars were added to the faceted browsing interface: (**A**) ‘Target category’ facet bar, (**B**) ‘Target of assay’ facet bar, (**C**) ‘Cell’ facet bar and (**D**) ‘Date range selector’ facet bar. The ‘Cell’ and ‘Target of assay’ facet bars that have long lists of values include the typeahead search feature to help users locate terms of interest more quickly.

The faceted browsing interface was updated to include new facet bars: ‘Cell’, ‘Target of assay’ and ‘Date range selection’. The ‘Cell’ facet bar helps to find experiments performed on specific cell type(s) and the ‘Target of assay’ facet bar helps to find experiments with a common target (e.g. transcription factor of interest). The ‘Date range selection’ facet bar allows the users to narrow down the time period in which experiments of interest became publicly available. Facet bars containing long lists of values (e.g. ‘Cell’ and ‘Target of assay’ facet bars) were added the ‘typeahead’ feature to facilitate the lookup for terms of interest (Figure 2).

Using data from NCBI Entrez Gene (https://www.ncbi.nlm.nih.gov/gene), HGNC (https://www.genenames.org/), MGNC (http://www.informatics.jax.org/mgihome/nomen/), FlyBase (https://flybase.org/), WormBase (https://www.wormbase.org) and the Gene Ontology Consortium (14–21), the DCC added Gene objects to the portal. Linkage of the target objects to the relevant gene objects allowed categorization of the targets based on the Gene Ontology annotations (for more details on the categorization method, visit https://www.encodeproject.org/target-categorization/). Target categories are listed under the ‘Target category’ facet bar (Figure 2A).

## DATA PROCESSING

Processing the vast amount of data that are hosted on the ENCODE portal is challenging. The variation between experimental results coming from different labs and performed using similar, but not identical protocols requires robust, production-grade uniform processing pipelines that ensure successful analyses and generate comparable outputs. The DCC is tasked with the development, implementation and execution of the uniform processing pipelines that are first defined by collaborative consortium working groups. Centralized processing using uniform processing pipelines by the DCC generates comparable analysis results free of technical artifacts arising from methodological variability across labs and time. All of the pipelines that are developed by the DCC are open source and are maintained in the DCC GitHub repository (Supplementary Table S2, https://github.com/ENCODE-DCC). The pipelines that have been developed in the previous funding phase of ENCODE have an implementation on the DNAnexus cloud platform (https://dnanexus.com), making them accessible to the larger scientific community for unrestricted use (3). The pipelines the DCC is working on in the current funding phase of ENCODE are being developed in a new framework that involves modern technologies and platforms such as Docker (https://www.docker.com), Singularity (https://sylabs.io/, 22), WDL (https://doi.org/10.7490/f1000research.1114631.1), CircleCI (https://circleci.com) and automated testing. The DCC has established this pipeline development framework to ensure reproducibility, portability and robustness of the new uniform processing pipelines.

## DATA VISUALIZATION

**Figure 3.**
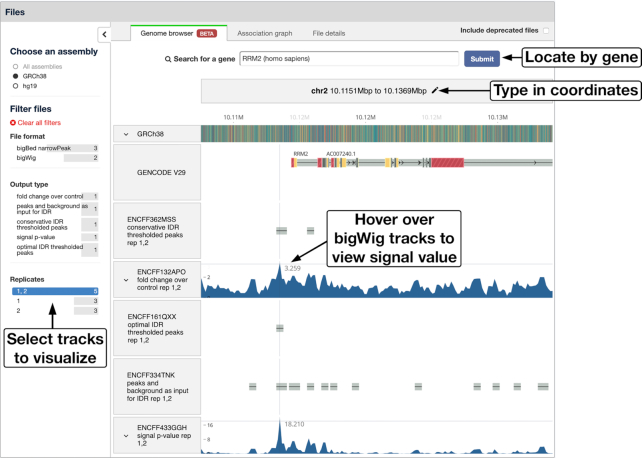
Valis genome browser embedded on the portal. Users can find their genomic region of interest by (i) searching for a specific gene, (ii) specifying genomic coordinates or (iii) using the mouse scrolling and zooming features directly to navigate the region. File selectors are available on the left, allowing users to select and visualize tracks for specific genome assembly, specific file format or output type, and files belonging to specific experimental replicate(s). This example can be found by clicking the ‘Genome browser’ tab on the experiment summary page (https://www.encodeproject.org/experiments/ENCSR807BGP/).

Data visualization is an essential step in the process of data analysis and interpretation. For the visualizable outputs of experiments on the ENCODE portal, the DCC has created tracks and track hubs, which can be visualized using the UCSC (https://genome.ucsc.edu/) and ENSEMBL (https://www.ensembl.org) genome browsers (23,24). These visualizations require redirection to the browser’s portal and the DCC has found that user experience is compromised by the limited track-related metadata that can be passed to the genome browser websites. To improve the user experience and provide independent data visualization capability, the DCC has embedded a GPU accelerated genome browser powered by Valis (https://valis.bio) on the ENCODE portal (Figure 3). Experiment pages on the portal have been redesigned, and the tab ‘Genome browser’ has been added for the embedded genome browser. The new ‘Genome browser’ tab allows users to inspect visualizable processed files, e.g. bigWig and bigBed file formats, deriving from the experiment of interest.

Genome browsers such as UCSC, ENSEMBL and Valis are designed to visualize genomic data along one dimension, the genomic position axis. The results of experiments probing the three-dimensional organization of the genome are difficult to visualize with these tools. The ENCODE consortium has generated data from a set of Hi-C, ChIA-PET and 5C experiments that characterize the three-dimensional chromatin organization in different cells and tissues (5,25–29). To support visualization of Hi-C results, portal pages for these experiments were modified to include links, which can be found on the ‘File details’ tab, redirecting to the Juicebox visualization software web page (https://aidenlab.org/juicebox/) and to allow selection of the relevant files for visualization (30,31).

## CART FOR CUSTOM COLLECTIONS OF DATASETS

**Figure 4.**
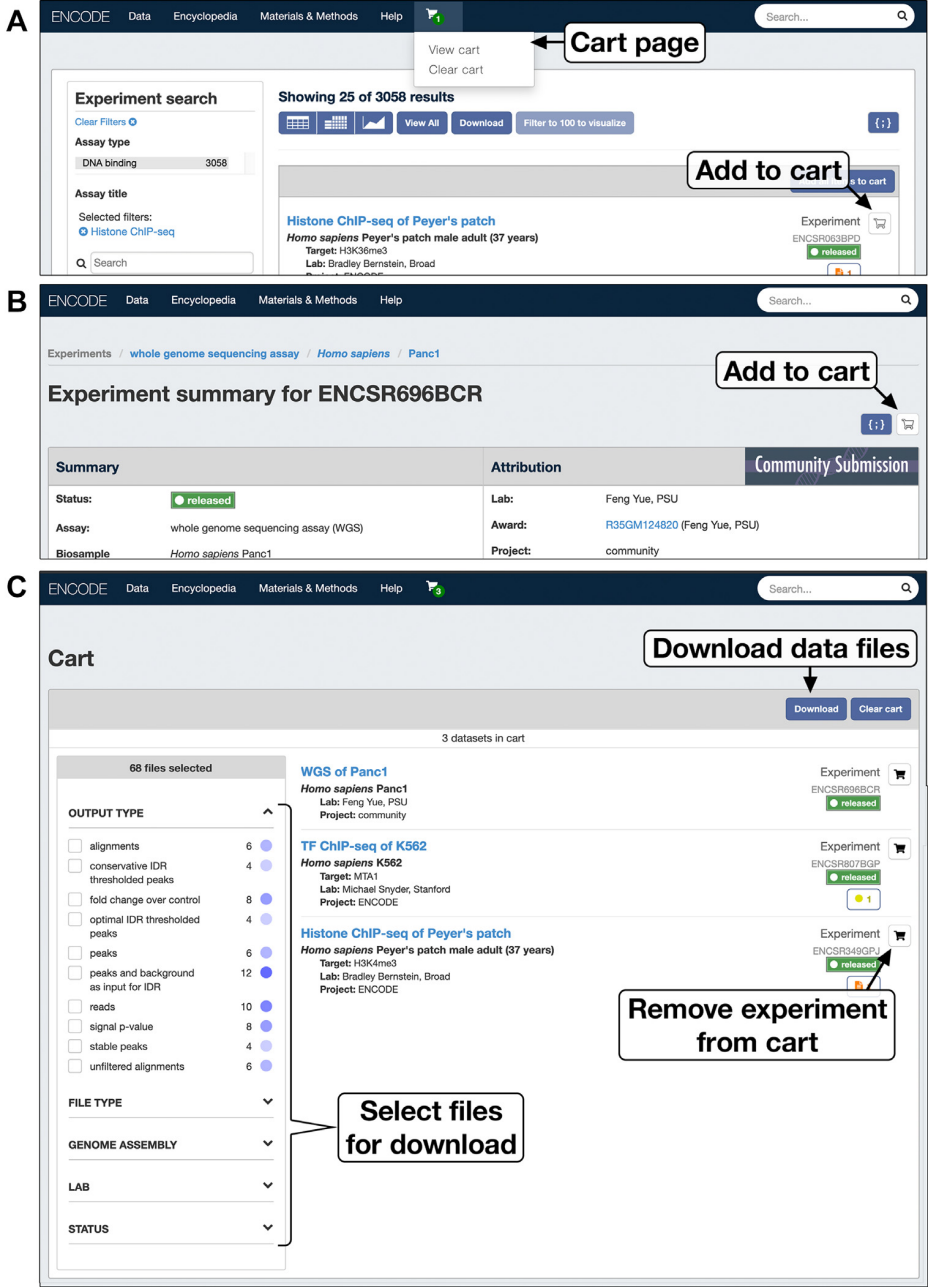
Cart for custom collections of datasets. Experiments can be added to the cart from the experiment search page **(A)** or by clicking on the cart icon on the experiment summary page **(B)**. Experiments that are no longer needed can be removed from the cart **(C)**. Files that belong to the experiments in the cart can be filtered using the file selectors on the left. For example, users can select only alignments mapped to the GRCh38 genome assembly. The cart allows users to download the data files satisfying specific criteria along with associated metadata. Currently, carts are saved per active browser session and will be erased after closing the web browser or refreshing the page.

The ENCODE portal has a free-text searching system and a faceted browsing interface that can be used to refine search queries on ENCODE metadata. Though these are powerful tools for finding datasets of interest, they do not support the creation of a custom experimental grouping. To address this need, DCC developed the cart feature that allows users to add one or more experiments into a cart to create an arbitrary collection of experiments. After building a cart-based data collection, users can filter and download data files along with the associated metadata related to the experiments from the cart (Figure 4).

## ENCODE AT AWS REGISTRY OF OPEN DATA

**Figure 5.**
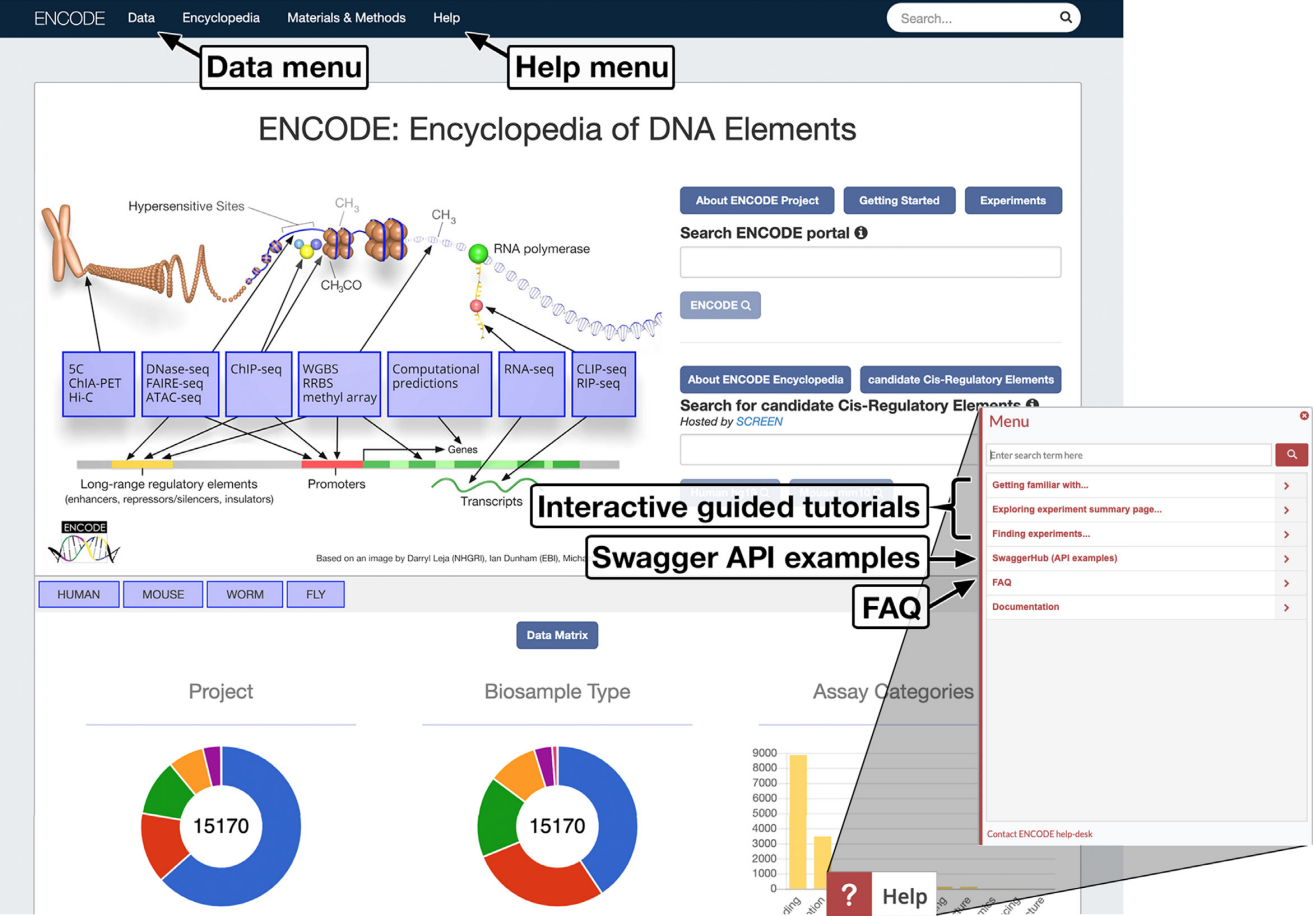
Tutorials, documentation and help on the ENCODE portal. Link to tutorials and examples for ENCODE AWS Registry of Open Data could be found under the ‘Data’ menu on the left-hand side of the top bar. Help information is accessible through the ‘Help’ menu on the top bar of every portal page. Interactive walk-through tutorials and additional help materials are available via the ‘Help’ widget visible in the bottom-right corner of the screen. The ENCODE DCC also supports users directly through the ENCODE help desk at encode-help@lists.stanford.edu.

The ENCODE portal provides open and unrestricted access to experimental metadata as well as the data files associated with the experiments. The data files accessible through the portal can be directly downloaded or accessed on the Amazon cloud. ENCODE has recently become part of the AWS Registry of Open Data and ENCODE data files are accessible via a public AWS Simple Storage Service (S3) bucket. The bucket is fully accessible for local cloud computing on AWS products. Alternatively, bucket content can be transferred like any other file. Using ENCODE data with AWS services is highly performant when data come directly from S3. The link to the ENCODE AWS Registry of Open Data page that includes tutorials and examples on ENCODE data use (https://registry.opendata.aws/encode-project/) can be found under the ‘Data’ menu on the ENCODE portal top toolbar (Figure 5).

## ENCODE PORTAL HELP AND DOCUMENTATION

All the experiments and their analyses on the ENCODE portal are represented using a structured metadata model that informs the interpretation of data in biological terms (3,4,6,32). The ENCODE portal includes new documentation and tutorials on how to use ENCODE metadata and data (https://www.encodeproject.org/help/getting-started/). In addition to FAQ and documentation web pages, a set of interactive tutorials was prepared using the WalkMe user onboarding platform (https://www.walkme.com/) and integrated on the portal web pages. Each tutorial guides users, step by step, toward a specific goal (Figure 5). The ENCODE portal also provides an API to facilitate programmatic access to metadata and data files. The API is documented using the Swagger platform with examples on the Swagger hub (https://app.swaggerhub.com/apis-docs/encodeproject/api/basic_search). The ‘Help’ rectangular widget located in the bottom-right corner of any page of the ENCODE portal provides access to the list of various interactive tutorials, FAQ, SwaggerHub and documentation pages (Figure 5).

## FUTURE DIRECTIONS

The ENCODE project is an ongoing collaborative research effort. For 16 years, ENCODE data have been used extensively by many researchers across the globe. The ENCODE consortium continues to provide unrestricted access to novel high-quality data for the scientific community. To more directly assess the function of ENCODE elements, new assays are underway, including massively parallel reporter assays (33), CRISPR screens (34) and STARR-seq (35). To increase the resolution of cell-type-specific states, new single-cell-based experimental assays are also underway. All of these new data will be made available through the ENCODE portal.

In addition to individual experiments and their metadata, the ENCODE portal also hosts results of integrative analysis such as the registry of cis-regulatory elements (manuscript submitted for publication) and other types of genome annotations computationally derived from experimental data coming from both the ENCODE consortium and the scientific community (https://www.encodeproject.org/matrix/?type=Annotation). The DCC will host and provide access to updated genome annotations generated by algorithms such as chromHMM (36) and Segway (37) using the rich and diverse data submitted to the ENCODE portal.

Genomic research is increasingly collaborative. The ENCODE consortium is an inherently collaborative project and extends this to collaborations with other consortia. For example, the ENCODE DCC shares epigenomic data with the International Human Epigenome Consortium (IHEC, http://ihec-epigenomes.org/). Together with IHEC, ENCODE initiated the EpiShare project, which is one of the driver projects of the Global Alliance for Genomics and Health (GA4GH, https://www.ga4gh.org/). The ENCODE portal infrastructure, metadata model and the uniform processing pipeline have been adopted by other projects and consortia. For example, the 4D Nucleome (4DN) (https://data.4dnucleome.org/) portal and Diabetes Epigenome Atlas (https://www.diabetesepigenome.org/) share the back-end SnoVault infrastructure developed and used for the ENCODE portal by the ENCODE DCC (38). IHEC and 4DN projects use uniform processing pipelines developed by the ENCODE DCC and the use of genome reference files required for data processing is being coordinated. The DCC continues to work closely with new consortia such as Human Cell Atlas and Human BioMolecular Atlas Program to create portable resources and interoperable data.

## DATA AVAILABILITY

ENCODE is an open source project with all software available in the GitHub repository (https://github.com/ENCODE-DCC).

## Supplementary Material

gkz1062_Supplemental_File

## Notes

Database URL: www.encodeproject.org
