# Supplementary material for: New developments on the Encyclopedia of DNA Elements (ENCODE) data portal
Source: Nucleic Acids Res. 2019 Nov 12;48(D1):D882–9. doi: 10.1093/nar/gkz1062 (PMC7061942; doi:10.1093/nar/gkz1062)
Supplement: gkz1062_Supplemental_File [file gkz1062_supplemental_file.docx]

**Supplementary Table S1.** New sample types added to the portal from 2017-11-01 to 2019-12-31

| **Biological sample type** | **Experiments** |
| --- | --- |
| ARPE-19 | <https://www.encodeproject.org/experiments/ENCSR110JOO/> |
| bone marrow-derived macrophage | https://www.encodeproject.org/experiments/ENCSR614KOV/,  https://www.encodeproject.org/experiments/ENCSR535SFV/,  https://www.encodeproject.org/experiments/ENCSR822FMG/,  https://www.encodeproject.org/experiments/ENCSR938LWT/,  https://www.encodeproject.org/experiments/ENCSR195JXZ/,  https://www.encodeproject.org/experiments/ENCSR879BNI/,  https://www.encodeproject.org/experiments/ENCSR614DLJ/,  https://www.encodeproject.org/experiments/ENCSR590GUK/,  https://www.encodeproject.org/experiments/ENCSR627RCT/,  https://www.encodeproject.org/experiments/ENCSR906KGC/,  https://www.encodeproject.org/experiments/ENCSR164XXE/,  https://www.encodeproject.org/experiments/ENCSR577EOP/,  https://www.encodeproject.org/experiments/ENCSR754DFU/,  https://www.encodeproject.org/experiments/ENCSR917CGF/,  https://www.encodeproject.org/experiments/ENCSR460HQN/,  https://www.encodeproject.org/experiments/ENCSR107TKJ/,  https://www.encodeproject.org/experiments/ENCSR959OQW/,  https://www.encodeproject.org/experiments/ENCSR596SMN/,  https://www.encodeproject.org/experiments/ENCSR156DUX/,  https://www.encodeproject.org/experiments/ENCSR803TDA/,  https://www.encodeproject.org/experiments/ENCSR731TGN/,  https://www.encodeproject.org/experiments/ENCSR504QOC/,  https://www.encodeproject.org/experiments/ENCSR464OLG/,  https://www.encodeproject.org/experiments/ENCSR814INP/,  https://www.encodeproject.org/experiments/ENCSR507OWX/ |
| cerebral cortex | https://www.encodeproject.org/experiments/ENCSR137GMB/,  https://www.encodeproject.org/experiments/ENCSR998UAE/,  https://www.encodeproject.org/experiments/ENCSR340GWV/,  https://www.encodeproject.org/experiments/ENCSR277BIL/ |
| DU 145 | <https://www.encodeproject.org/experiments/ENCSR672RHL/> |
| ecto neural progenitor cell | <https://www.encodeproject.org/experiments/ENCSR360XIS/> |
| femur | <https://www.encodeproject.org/experiments/ENCSR805XIF/> |
| GM20431 | <https://www.encodeproject.org/experiments/ENCSR118YWJ/> |
| HCEC 1CT | <https://www.encodeproject.org/experiments/ENCSR038XTK/> |
| HFFc6 | https://www.encodeproject.org/experiments/ENCSR519CMW/,  https://www.encodeproject.org/experiments/ENCSR510VXV/,  https://www.encodeproject.org/experiments/ENCSR114FEG/,  https://www.encodeproject.org/experiments/ENCSR639PCR/,  https://www.encodeproject.org/experiments/ENCSR902GAF/,  https://www.encodeproject.org/experiments/ENCSR750ZGO/,  https://www.encodeproject.org/experiments/ENCSR672EWY/,  https://www.encodeproject.org/experiments/ENCSR647WPA/ |
| HK-2 | <https://www.encodeproject.org/experiments/ENCSR634YVQ/> |
| HT-1197 | <https://www.encodeproject.org/experiments/ENCSR386KHY/> |
| HT-1376 | <https://www.encodeproject.org/experiments/ENCSR933UZH/> |
| hTERT-HME1 | <https://www.encodeproject.org/experiments/ENCSR991JXX/> |
| K1 | <https://www.encodeproject.org/experiments/ENCSR314HAC/> |
| kidney capillary endothelial cell | <https://www.encodeproject.org/experiments/ENCSR988YKR/> |
| kidney glomerular epithelial cell | <https://www.encodeproject.org/experiments/ENCSR136KEL/> |
| kidney tubule cell | https://www.encodeproject.org/experiments/ENCSR518ZJY/,  https://www.encodeproject.org/experiments/ENCSR659ERI/,  https://www.encodeproject.org/experiments/ENCSR657XJU/,  https://www.encodeproject.org/experiments/ENCSR257CIZ/,  https://www.encodeproject.org/experiments/ENCSR332ZJL/,  https://www.encodeproject.org/experiments/ENCSR175IWT/ |
| KU-19-19 | <https://www.encodeproject.org/experiments/ENCSR404HWQ/> |
| left arm bone | <https://www.encodeproject.org/experiments/ENCSR976XOY/> |
| left leg bone | <https://www.encodeproject.org/experiments/ENCSR431UEM/> |
| mature B cell | https://www.encodeproject.org/experiments/ENCSR012UKZ/,  https://www.encodeproject.org/experiments/ENCSR613GYI/,  https://www.encodeproject.org/experiments/ENCSR690BTJ/,  https://www.encodeproject.org/experiments/ENCSR847UWT/,  https://www.encodeproject.org/experiments/ENCSR753DHV/,  https://www.encodeproject.org/experiments/ENCSR870DKF/,  https://www.encodeproject.org/experiments/ENCSR471ZWB/,  https://www.encodeproject.org/experiments/ENCSR407TGO/ |
| monocyte | https://www.encodeproject.org/experiments/ENCSR862JVD/,  https://www.encodeproject.org/experiments/ENCSR069PIG/ |
| mouse embryonic stem cell | https://www.encodeproject.org/experiments/ENCSR028GRK/,  https://www.encodeproject.org/experiments/ENCSR298WSI/ |
| MSiPS | <https://www.encodeproject.org/experiments/ENCSR778FXH/> |
| MSLCL | <https://www.encodeproject.org/experiments/ENCSR452NHL/> |
| NCI-H1437 | <https://www.encodeproject.org/experiments/ENCSR833CMG/> |
| parathyroid adenoma | https://www.encodeproject.org/experiments/ENCSR486GER/,  https://www.encodeproject.org/experiments/ENCSR086FIZ/,  https://www.encodeproject.org/experiments/ENCSR336PTS/,  https://www.encodeproject.org/experiments/ENCSR335LTZ/,  https://www.encodeproject.org/experiments/ENCSR249LNW/,  https://www.encodeproject.org/experiments/ENCSR034RQV/,  https://www.encodeproject.org/experiments/ENCSR168STG/,  https://www.encodeproject.org/experiments/ENCSR404IRS/,  https://www.encodeproject.org/experiments/ENCSR780OZE/,  https://www.encodeproject.org/experiments/ENCSR688SJY/,  https://www.encodeproject.org/experiments/ENCSR033OKS/,  https://www.encodeproject.org/experiments/ENCSR619AAK/,  https://www.encodeproject.org/experiments/ENCSR256KRN/,  https://www.encodeproject.org/experiments/ENCSR370KYM/,  https://www.encodeproject.org/experiments/ENCSR481IYI/,  https://www.encodeproject.org/experiments/ENCSR434NUA/,  https://www.encodeproject.org/experiments/ENCSR106GXJ/,  https://www.encodeproject.org/experiments/ENCSR492DBQ/,  https://www.encodeproject.org/experiments/ENCSR735SLW/ |
| right arm bone | <https://www.encodeproject.org/experiments/ENCSR274SDO/> |
| right leg bone | <https://www.encodeproject.org/experiments/ENCSR449HOQ/> |
| SU-DHL-4 | <https://www.encodeproject.org/experiments/ENCSR658RQQ/> |
| THP-1 | https://www.encodeproject.org/experiments/ENCSR748LQF/,  https://www.encodeproject.org/experiments/ENCSR669GJD/ |
| yolk sac | <https://www.encodeproject.org/experiments/ENCSR894HWV/> |

Experiments in this table are a subset of over 2000 new experiments added to the portal between 2017-11-01 and 2019-12-31. All the experiments added in this period could be accessed using the following URL: [https://www.encodeproject.org/matrix/?type=Experiment&status=released&advancedQuery=@type:Experiment+date_released:[2017-11-01+TO+2019-12-31]](https://www.encodeproject.org/matrix/?type=Experiment&status=released&advancedQuery=@type:Experiment+date_released:%5B2017-11-01+TO+2019-12-31%5D)

**Supplementary Table S2.** DCC GitHub repositories for ENCODE uniform data processing pipelines

|  | Uniform processing pipeline | GitHub repository |
| --- | --- | --- |
| Uniform processing pipelines utilized for data processing primarily in the previous ENCODE phase and available on DNAnexus | ChIP-seq | <https://github.com/ENCODE-DCC/chip-seq-pipeline> |
|  | bulk RNA-seq | <https://github.com/ENCODE-DCC/long-rna-seq-pipeline> |
|  | DNase-seq | <https://github.com/ENCODE-DCC/dnase_pipeline> |
|  | WGBS | <https://github.com/ENCODE-DCC/dna-me-pipeline> |
| New WDL based uniform processing pipelines. Pipelines marked with * are currently in different stages of development. | ChIP-seq | <https://github.com/ENCODE-DCC/chip-seq-pipeline2> |
|  | ATAC-seq^*^ | <https://github.com/ENCODE-DCC/atac-seq-pipeline> |
|  | bulk RNA-seq^*^ | <https://github.com/ENCODE-DCC/rna-seq-pipeline> |
|  | DNase-seq^*^ | <https://github.com/ENCODE-DCC/dnase-seq-pipeline> |
|  | WGBS^*^ | <https://github.com/ENCODE-DCC/wgbs-pipeline> |
|  | microRNA-seq | <https://github.com/ENCODE-DCC/mirna-seq-pipeline> |
|  | long read RNA-seq^*^ | <https://github.com/ENCODE-DCC/long-read-rna-pipeline> |
|  | HiC^*^ | <https://github.com/ENCODE-DCC/hic-pipeline> |
